# Supplementary material for: Cytological and genetic consequences for the progeny of a mitotic catastrophe provoked by Topoisomerase II deficiency
Source: Aging (Albany NY). 2019 Dec 8;11(23):11686–721. doi: 10.18632/aging.102573 (PMC6932922; doi:10.18632/aging.102573)
Supplement: Supplementary Materials [file aging-11-102573-s001..pdf]

SUPPLEMENTARY MATERIALS

Interpretation of microcolony assays

Cells from asynchronous cultures were seeded on the surface of a plate at a cell density that optimized distance between cells and number of cells per field. Selected fields normally contain budded ($S/G_2/M$) and unbudded (G_0/G_1) cells, oscillating from a 1:1 to a 1:2 ratio. In our analyses, we concentrated on unbudded cells. A picture was taken before shifting the incubation temperature to 37 ºC (0h), then another picture of the same field was taken after the 37 ºC incubation (either 6h or 24h), and a final third picture was taken the day after, once the plate had been re-incubated at 25 ºC. The situation of each G_0/G_1 cell at the beginning of the assay was monitored after the 37 ºC incubations (6h or 24h) and after the plates were shifted back to 25 ºC. The landscape of possible outcomes is as shown in the Supplementary Table 5 (indicating "cell bodies" that may raise from the one-bodied G_0/G_1 cell). Each G_1/G_0 cells could travel through any of the indicated categories. In the main text, we sometimes refer to their trajectory as, for example, 2 → 1. This indicates that one G_1/G_0 at 0h became 2 bodies after the 37 ºC incubation, but then one body lysed, and the other did not bud again despite reactivating Top2 (25 ºC). Likewise, 3 → m means that the G_1/G_0 cell became a triplet at 37 ºC and then formed a large microcolony after Top2 re-activation. Because the quantification of all trajectories is complex, as shown in the outcome landscape table, we opted for sunburst charts (Figures 2B, 2F, 3E, 4A). The inner circle in the sunburst chart depicts proportions of cell bodies after the 37 ºC incubation. The outer circle depicts proportion of cell bodies after the final 25 ºC incubations for each situation observed in the inner circle. In order to aid with the interpretation of trajectories, we used different colors for different cell body numbers in the inner circle, but kept the colors of the inner circle for the same progeny in the outer circle. The changes in body numbers between the inner and the outer circles are indicated by numbers.

Interpretation of SNP arrays to detect genome rearrangements

Each SNP array contains 25-base oligonucleotides that matched either the W303-1A-associated allele or the YJM789-associated allele for about 13,000 different SNPs (out of 55,000 SNPs). The selected SNPs are distributed evenly across the genome, giving an array resolution of ~1 Kb (yeast genome is 13 Mb, excluding the 1-2 Mb of the repetitive ribosomal DNA array). By measuring the relative amounts of hybridization to each oligonucleotide, we could detect loss of heterozygosity (LOH) (an event expected from mitotic recombination), as well as analyse deletions, duplications, and changes in chromosome number. Examples of these types of genome rearrangements are given in the plots shown in Figure 5E and Supplementary Figure 8. The Y-axis shows the normalized hybridization ratio to probes specific to the W303-1A form of the SNP (red) or the YJM789 form of the SNP (blue). Heterozygous SNPs have ratios of about 1; in LOH events, SNPs derived from one homolog have a ratio of near 2 and those derived from the other have a ratio near 0. One common pattern is a terminal LOH (T-LOH) event, which can reflect either a reciprocal crossover or a non-reciprocal type of recombination termed "break-induced replication" (BIR, Figure 5E) [1]. A second type of LOH is an interstitial LOH event (I-LOH) in which a region of LOH is flanked by heterozygous. I-LOH events (gene conversions) result from the non-reciprocal transfer of DNA sequences between homologs. Two other classes of genomic rearrangements are a consequence of chromosome non-disjunction. Such non-disjunction events can result in trisomy or monosomy. An event in which one homolog is duplicated and another deleted is called "uniparental disomy" (UPD) and can reflect a non-disjunction event in which the two homologs segregate into different daughter cells.

MATERIALS AND METHODS

Construction of the *top2-5/top2-5* isogenic homozygous diploid (FM1730)

We employed the one-step marker-free transformation-based protocol described in Supplementary Figure 9. Briefly, the haploid *MAT*a *bar1Δ top2-5 HTA2-GFP* strain used in most experiments (FM1386) was transformed with a PCR product obtained from a *MAT***α** haploid strain. This product is designed such that it can only recombine with the *MAT* locus but not with the silent *HML/HMR* loci. We counterselected against the *MAT*a genotype by spreading 5 µg α-factor on the Petri dish surface before spreading the transformed cells. Colonies resistant to α-factor were collected after 3-4 days at 25 ºC and checked by PCR for the *MAT*a, *MAT*α or *MAT*a/*MAT*α genotypes (Supplementary Figure 9). *MAT*a/*MAT*α diploids were further confirmed by sporulation capability and 2N DNA content by flow cytometry [2].

Construction of the *top2-5/top2-5* hybrid heterozygous diploids (FM1873 and MD684)

The *top2-5* heterozygous diploids FM1873 and MD684 were obtained by crossing of haploid strain

top2-5 derivatives of PSL2 and PSL5. These two strains are isogenic with W303-1A and YJM789, respectively, and have been engineered to select and visually detect chromosome V rearrangements [3]. W303-1A and YJM789 differ by about 55,000 SNPs. The PSL2 *top2-5* (FM1830) and PSL5 *top2-5* (FM1832) haploids were constructed by transformation with a *top2-5:9xmyc:natNT2* product. This product was amplified by PCR from a CH326 strain derivative in which the *top2-5* allele had been tagged at 3' with sequences for 9 copies of the Myc epitope [4]. The heterozygous *top2-5/top2-5* diploid FM1873 was obtained by crossing FM1830 and FM1832. After realizing that FM1873 already carried genome alterations at 25 ºC (3-4 copies of cXIV and cXIIr T-LOH), other attempts to construct this diploid were undertaken. Previously, the FM1830 and FM1832 haploids were analysed and it was determined that FM1830 had 1-2 copies of cXIV. Thus, FM1830 was backcrossed with W303 to cure the strain of genome alterations. One spore (MD681) was identified that had the same genotype as FM1830 except that it had only one copy of chromosome XIV. This strain was crossed to FM1832 to generate the diploid MD684. Although these steps of construction were designed to generate a diploid that was isogenic with FM1873 lacking the aneuploidy of XIV and the T-LOH event on cXIIr, subsequent microarray analysis showed that MD684 still had three to four copies of XIV, although the T-LOH event on cXIIr was absent.

Clonogenic survivability assays

Clonogenic assays were performed directly on agar plates to determine survivability of the progeny regardless the actual number of daughter cells originated at the restrictive condition. For this purpose, 10^2 and 10^3 cells (as estimated after counting cells in an asynchronous logarithmic culture with a Neubauer chamber) were spread onto a set of 14 YPD plates. These plates were then incubated for 0, 3, 6, 9, 12, 24 and 48 h at 37 ºC. After that, they were switched to 25 ºC to allow the growth of the survivors. Colonies were counted after 3–4 days and were normalized to the number of colonies grown without exposure to the restrictive temperature (0 h).

Microcolony assays

For the microcolony analysis, ~1.5×10^5 cells (counted by a Neubauer chamber) were spread onto a YPD (or YPD plus 1.2 M Sorbitol) plate to yield a density on the plate surface of around 25 cells per 10,000 μm^2. Defined positions on the plate were marked by piercing the surface with the needle of a Singer Sporeplay tetrad microdissector, using its 8 × 8 grid as a reference (12-16 fields in total). The plate was then transferred to a Leica LMD6000 direct microscope equipped with a 6.7x and 40x long-range objectives. The 6.7x was used to locate the marked fields and the 40x to take pictures of the cells in those fields (corresponding to 0 h, 25 ºC). Next, the plates were incubated at 37 ºC for 6 or 24 h before taking new pictures of the same fields. The procedure was repeated one more time after incubating the plate back at 25 ºC for 18–24 h. Finally, the same microcolonies in the corresponding three frames per field were identified by eye and categorized as indicated in the figure legends.

Single cell analysis of the progeny by micromanipulation

For micromanipulation of the progeny the different strains were streaked on YPD Petri dishes. Unbudded cells were harvested with a Singer Sporeplay tetrad microdissector. Just 12 cells were harvested per plate and arrayed along the A file stage grid template in order to avoid prolonged incubations at 25º C. They were then incubated 6 h at 37 ºC and then observed under the microscope to count the number of cells that originated from the initial cell. Next, each cell that had at least a new bud was subjected to a mild attempt to separate the cell bodies using the needle and the vibration device. If successful, the largest body remained in the A file, whereas the other body was transferred to the B file. Finally, the plate was incubated 4 d at 25 ºC to search for survivors.

Assays to determine segregation and morphology of the nucleus

The segregation and morphology of the histone-labelled nucleus (H2A-GFP) was analyzed by wide-field fluorescence videomicroscopy. An asynchronous culture was concentrated by centrifugation to OD_{660} of 3 and spread onto YPD agar 90 mm Petri dishes. Patches were made from this plate and mounted on a microscope slide. They were incubated at 37 ºC for 24 h in high humidity chambers to avoid the patch to dry. The same fields were photomicrographed at 0, 6 and 24 h, or as reported before [5]. For each time point, a series of z-focal plane images (10 planes, 0.6 µm depth) were collected on a Leica DMI6000, using a 63x/1.30 immersion objective and an ultrasensitive DFC 350 digital camera, and processed with the AF6000 software (Leica).

SNP microarrays

In brief, for most experiments, genomic DNA was obtained from single-colony isolates of experimental

samples (incubated for six hours at 37 ºC) that was labeled with Cy5-dUTP. Two different types of microarray-control DNA were used and labelled with Cy3-dUTP. For some experiments, we used DNA purified from the FM1873 culture before exposure to 37 ºC. In other experiments, we used DNA from the *TOP2/TOP2* isogenic strain JSC24 [1]. Following labeling of the samples, the experimental and microarray-control DNA samples were mixed and hybridized to the microarrays [6]. The microarrays were then scanned at wavelengths 532 and 635 nm with a GenePix scanner, and analyzed by GenePix Pro software. Hybridization signals for Cy5 and Cy3 were normalized over the array to a value of 1. Additional steps of normalization are described in [6]. Following normalization, the ratio of hybridization of the experimental samples to the control samples for individual SNPs was 1 if the experimental strain was heterozygous.

REFERENCES

1. Yin Y, Petes TD. Genome-Wide High-Resolution Mapping of UV-Induced Mitotic Recombination Events in Saccharomyces cerevisiae. PLoS Genet. 2013; 9. https://doi.org/10.1371/journal.pgen.1003894 PMID:24204306

2. García-Luis J, Machín F. Mus81-Mms4 and Yen1 resolve a novel anaphase bridge formed by noncanonical Holliday junctions. Nat Commun. 2014; 5: 5652. https://doi.org/10.1038/ncomms6652 PMID:25466415

3. Lee PS, Greenwell PW, Dominska M, Gawel M, Hamilton M, Petes TD. A fine-structure map of spontaneous mitotic crossovers in the yeast Saccharomyces cerevisiae. PLoS Genet. 2009; 5: e1000410. https://doi.org/10.1371/journal.pgen.1000410 PMID:19282969

4. Janke C, Magiera MM, Rathfelder N, Taxis C, Reber S, Maekawa H, Moreno-Borchart A, Doenges G, Schwob E, Schiebel E, Knop M. A versatile toolbox for PCR-based tagging of yeast genes: new fluorescent proteins, more markers and promoter substitution cassettes. Yeast. 2004; 21:947–62. https://doi.org/10.1002/yea.1142 PMID:15334558

5. Ramos-Pérez C, Ayra-Plasencia J, Matos-Perdomo E, Lisby M, Brown GW, Machín F. Genome-Scale Genetic Interactions and Cell Imaging Confirm Cytokinesis as Deleterious to Transient Topoisomerase II Deficiency in Saccharomyces cerevisiae. G3 (Bethesda). 2017; 7:3379–91. https://doi.org/10.1534/g3.117.300104 PMID:28839115

6. St. Charles J, Hazkani-Covo E, Yin Y, Andersen SL, Dietrich FS, Greenwell PW, Malc E, Mieczkowski P, Petes TD. High-resolution genome-wide analysis of irradiated (UV and γ-Rays) diploid yeast cells reveals a high frequency of genomic loss of heterozygosity (LOH) events. Genetics. 2012; 190:1267–84. https://doi.org/10.1534/genetics.111.137927 PMID:22267500

7. Haber JE. Mating-type gene switching in Saccharomyces cerevisiae. Annu Rev Genet. 1998; 32:561–99. https://doi.org/10.1146/annurev.genet.32.1.561 PMID:9928492

8. Holm C, Goto T, Wang JC, Botstein D. DNA topoisomerase II is required at the time of mitosis in yeast. Cell. 1985; 41:553–63. https://doi.org/10.1016/S0092-8674(85)80028-3
