# Supplementary material for: Cytological and genetic consequences for the progeny of a mitotic catastrophe provoked by Topoisomerase II deficiency
Source: Aging (Albany NY). 2019 Dec 8;11(23):11686–721. doi: 10.18632/aging.102573 (PMC6932922; doi:10.18632/aging.102573)
Supplement: Supplementary Figures [file aging-11-102573-s003..pdf]

SUPPLEMENTARY FIGURES

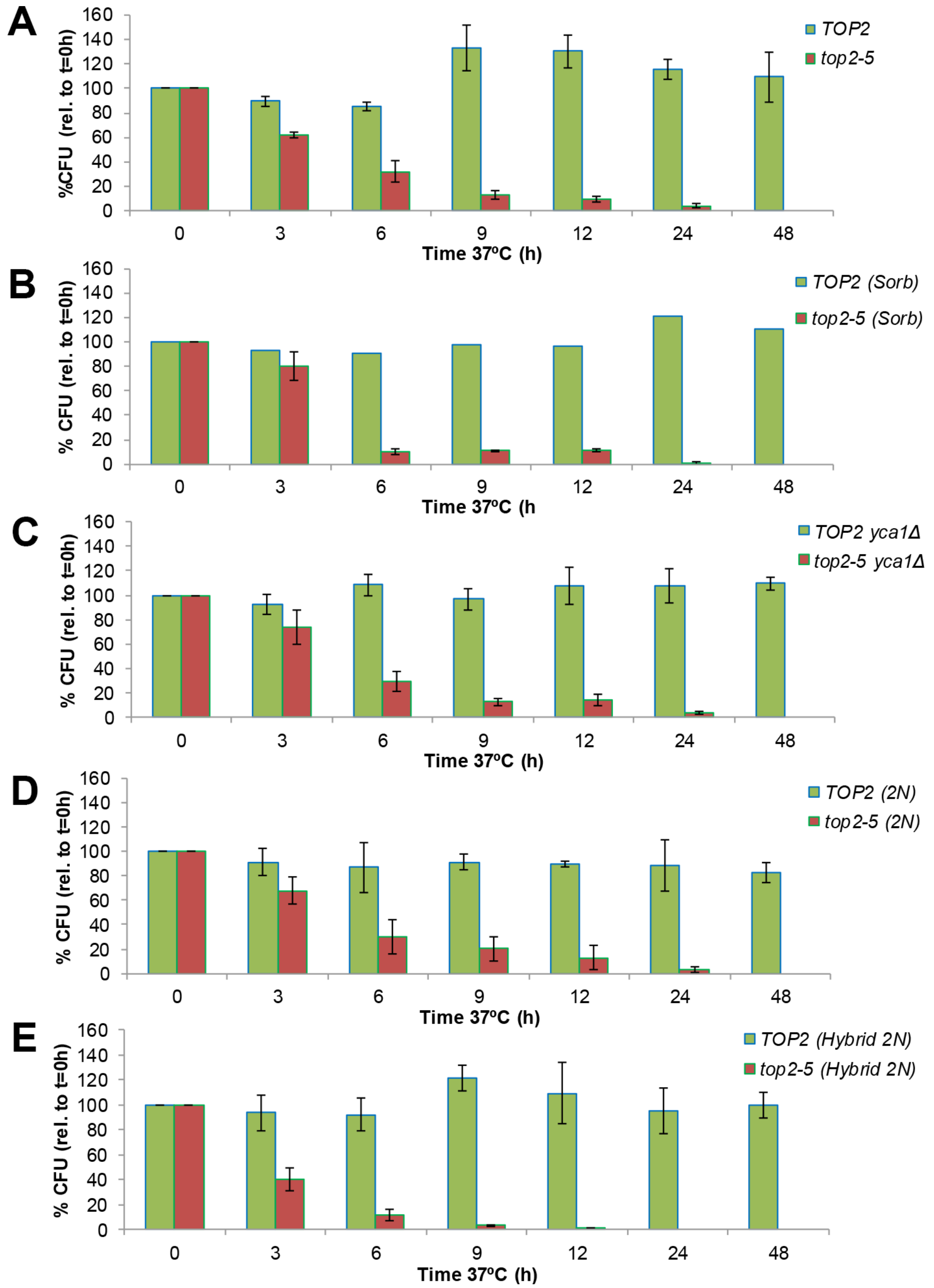

Supplementary Figure 1. Time course of clonogenic survivability of all *top2-5* strains and conditions studied and comparisons with their *TOP2* counterparts. Asynchronous cultures growing at 25 ºC were spread onto several plates for each strain or condition (mean +/- sem, n=3; except for TOP2 strain in Sorbitol where n=1). The plates were incubated at 37 ºC for different periods before transferring them 25 ºC. Four days after the initial plating, visible colonies (macrocolonies) were counted and normalized to a control plate which was never incubated at 37 ºC (0h). (**A**) haploid *top2-5* (FM1386) vs haploid *TOP2* (FM1419) on YPD. (**B**) The same two strains on YPD plus 1.2 M Sorbitol. (**C**) haploid *top2-5 yca1*Δ (FM1856) vs haploid *TOP2 yca1*Δ (FM1871) on YPD. (**D**) Homozygous diploid *top2-5* (FM1730) vs homozygous diploid *TOP2* (FM1732) on YPD. (**E**) Hybrid heterozygous diploid *top2-5* (FM1873) vs hybrid heterozygous diploid *TOP2* (FM2010) on YPD. These charts are related to Figures 1D, 2E, 3D, 4B and 5C, respectively.

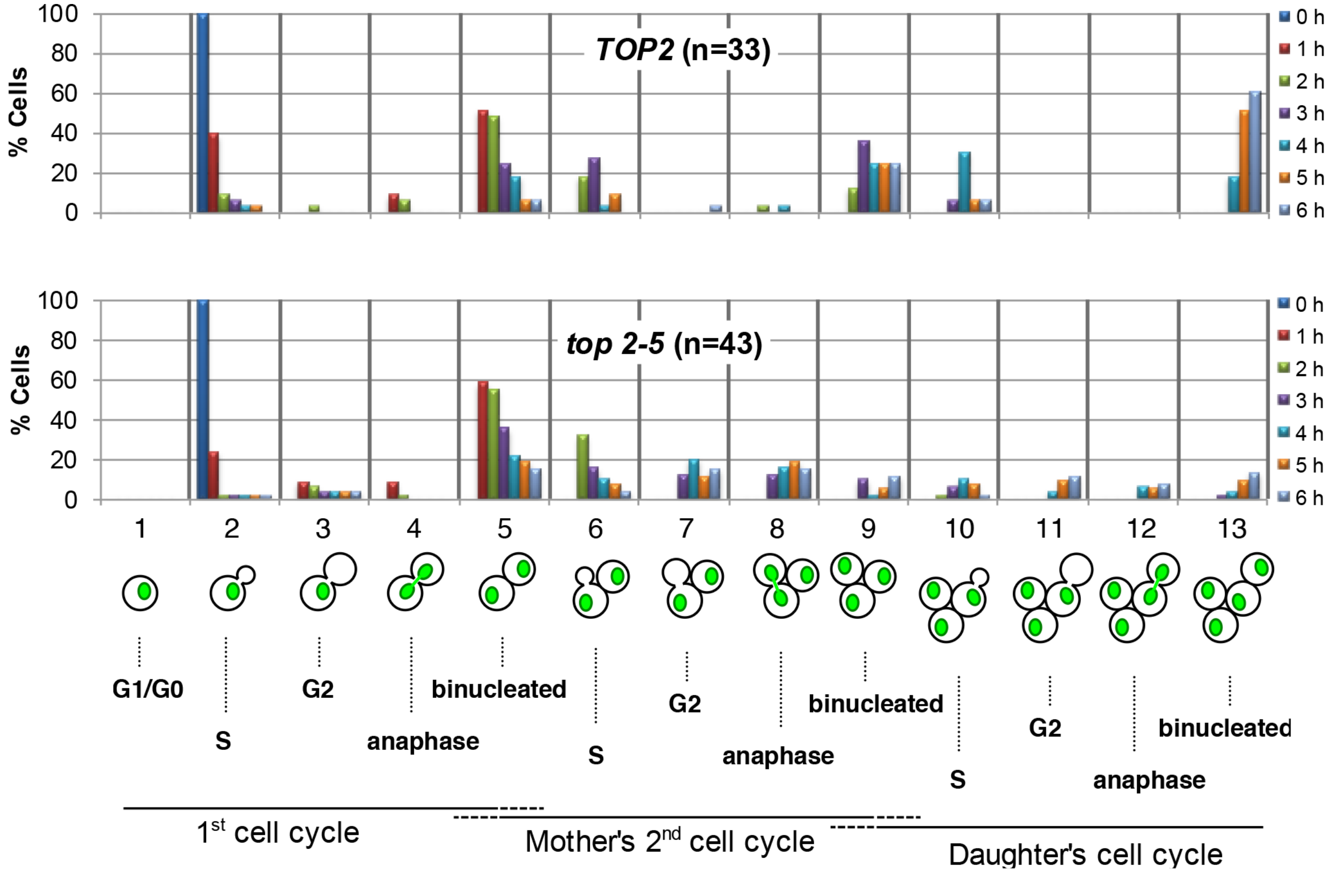

Supplementary Figure 2. Progression of cell division in budded *top2-5* cells at the time of the 37 ºC upshift. HTA2-GFP cells carrying either the *TOP2* or the *top2-5* alleles were grown at 25 ºC concentrated to OD_{600}=3, spread onto YPD agarose patches and filmed under the microscope in a 37 ºC incubation. Total number of cells analysed (N) is indicated for each chart. Each hour, during a period of 6 h, a new photo was taken and each cell was moved to one of the indicated categories depending on whether it has budded, re-budded, segregated or attempted to split the nuclear mass, and whether any of these events have occurred in the mother or the daughter cell coming from the first division. The cells analysed in this experiment come from the same fields in which only unbudded cells at 0 h were followed in the Figure 2A in [5]. In this case, only budded cells at 0 h were considered.

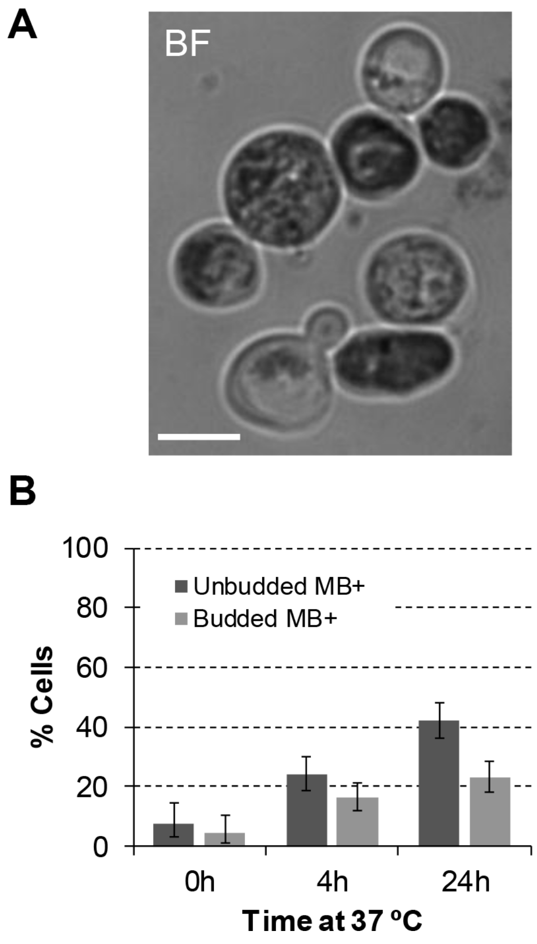

Supplementary Figure 3. Distribution of methylene blue staining in the *top2-5* population after incubation at 37 ºC for 24 h. Related to Figure 3B. (**A**) Examples of cells that stained or not blue (dark grey in the image) after 24 h at 37º C. Note at the bottom of the image a triplet (mother with two attached daughters) where only one cell body (first daughter) has lost vitality. Scale bar depicts 5 μm; BF, bright field. (**B**) Percentage of MB+ cells at 0, 4 and 24 h at 37 ºC (± CI95). Cells were firstly classified as unbudded or budded (2-3 cell bodies). Budded cells were scored as MB+ if at least one body stained positive. The unbudded:budded ratio was ~2:1 in this experiment. Thus, the 40%:20% ratio after 24 h at 37 ºC implies that unbudded and budded cells are equally stained in relative terms.

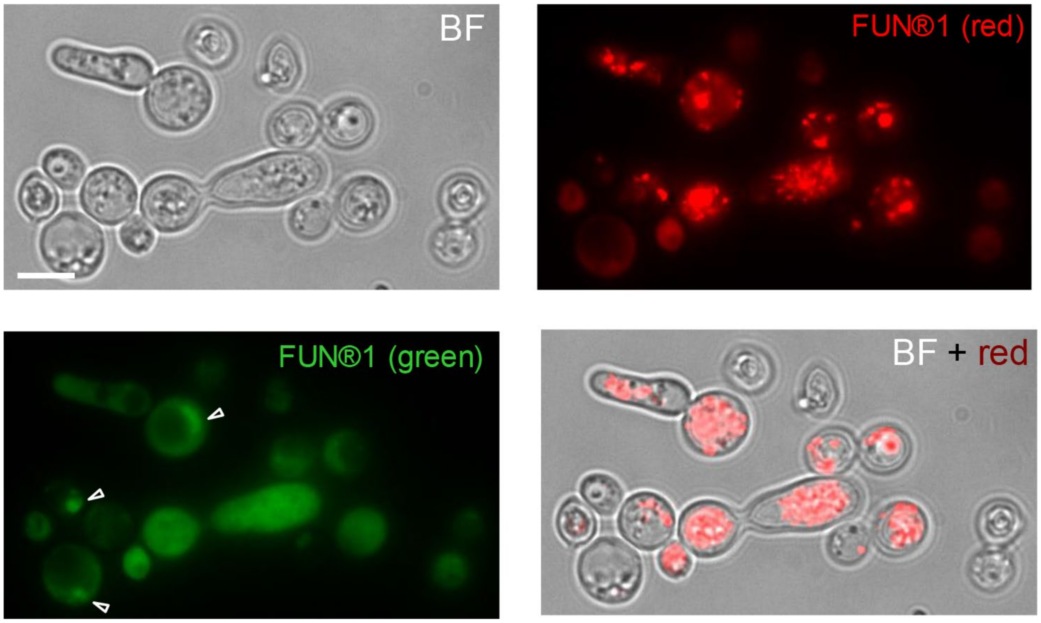

Supplementary Figure 4. Examples of *top2-5* cells stained with the vitality marker FUN® 1 after 24 h at 37 °C. Related to Figure 3C. In the green channel, hollow arrowheads point to the H2A-GFP signal. The cytoplasmic signal comes from unmetabolized FUN® 1. The brilliant aggregates in the red channel are metabolized FUN® 1 in cell bodies that retained a high vitality. Scale bar depicts 5 μm; BF, bright field.

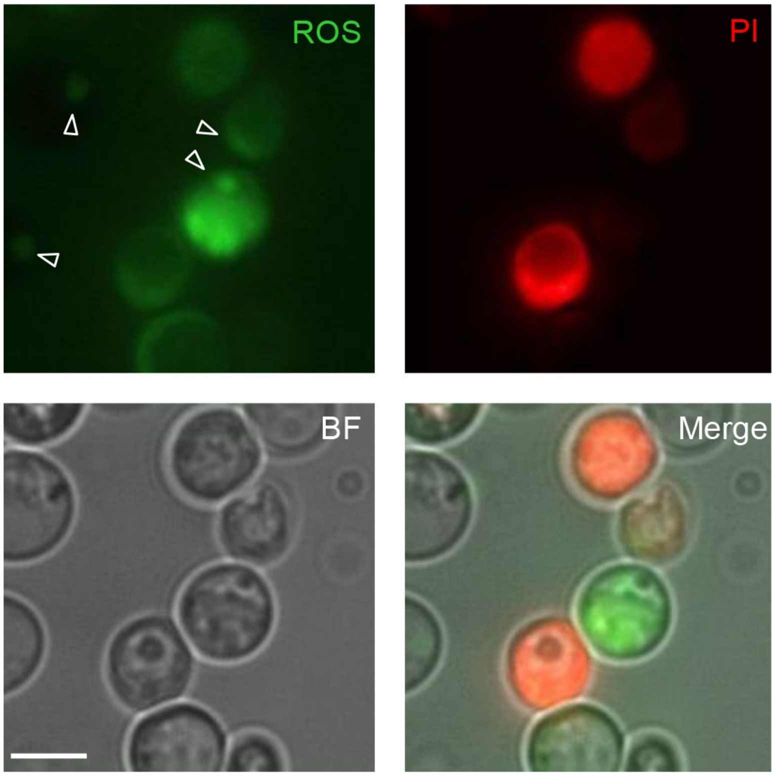

Supplementary Figure 5. Examples of *top2-5* cells co-stained for ROS and death after 24 h at 37 °C. Related to Figure 3C. In the green channel, hollow arrowheads point to the H2A-GFP signal. The cytoplasmic signal comes from ROS as reported with the DCFH-CA marker. The PI marker only stain cells that have lost plasma membrane impermeability (i.e., death cells). Note that cells are classified as ROS+ if they are bright green and PI negative. Scale bar depicts 5 μm; BF, bright field.

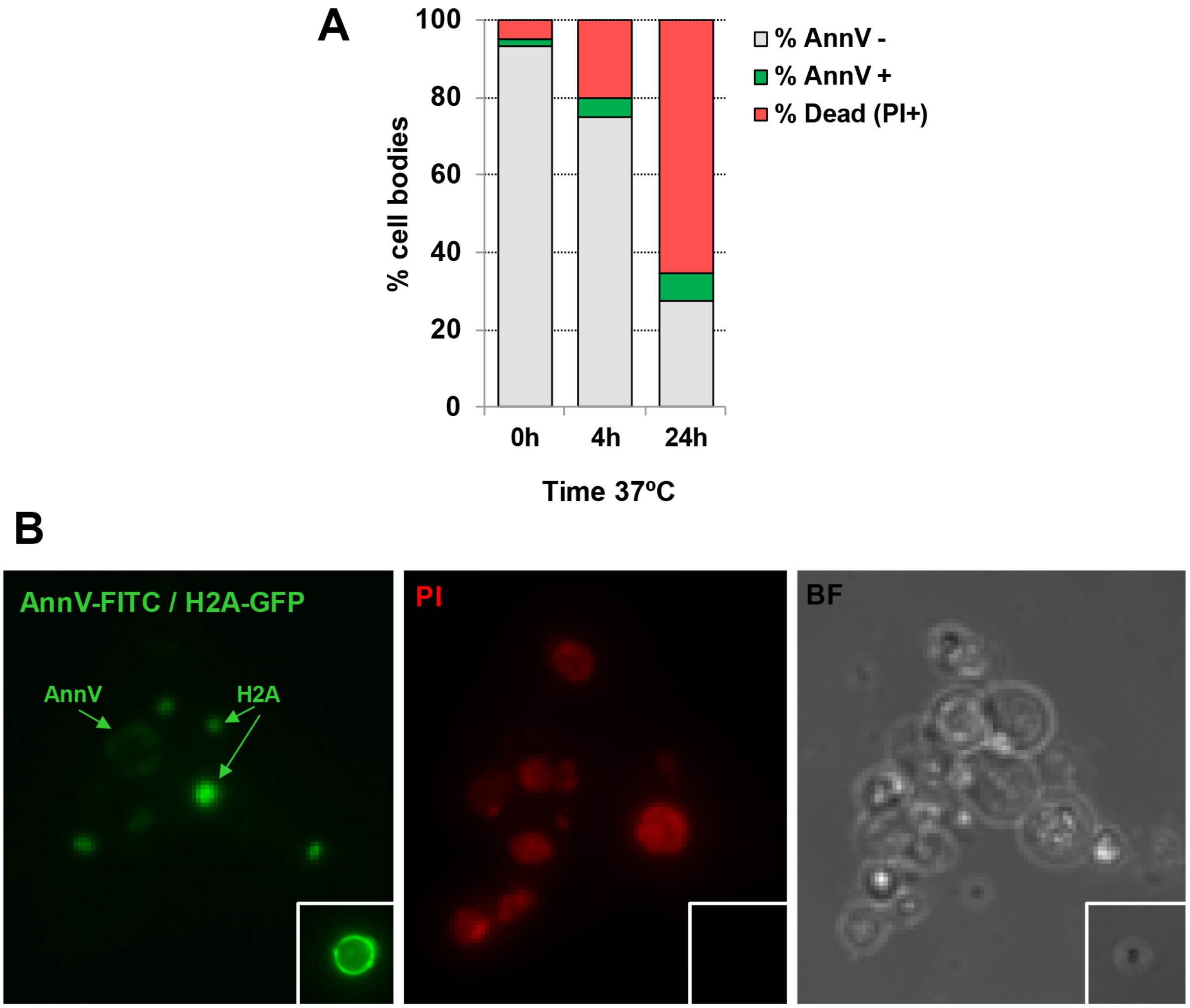

Supplementary Figure 6. Distribution of annexin V (annV) and propidium iodide (PI) co-staining in the *top2-5* population after incubation at 37 ºC. (**A**) Percentage of cell bodies with different co-staining patterns at 0, 4 and 24 h at 37 ºC. (**B**) Examples of cell bodies with different co-staining patterns at 24 h. Note that cells also carry nuclear H2A labelled with GFP. Cell bodies were classified as AnnV+ when the fluorescent green signal was seen at the body periphery (as indicated). The inlet field depicts a cell membranous subparticle (probably an organelle) strongly stained by annexin V-FITC. These unusual examples (<1% of "bodies") were not considered in the quantification since a proper cell body was not inferred in the BF. Scale bar depicts 5 µm; BF, bright field.

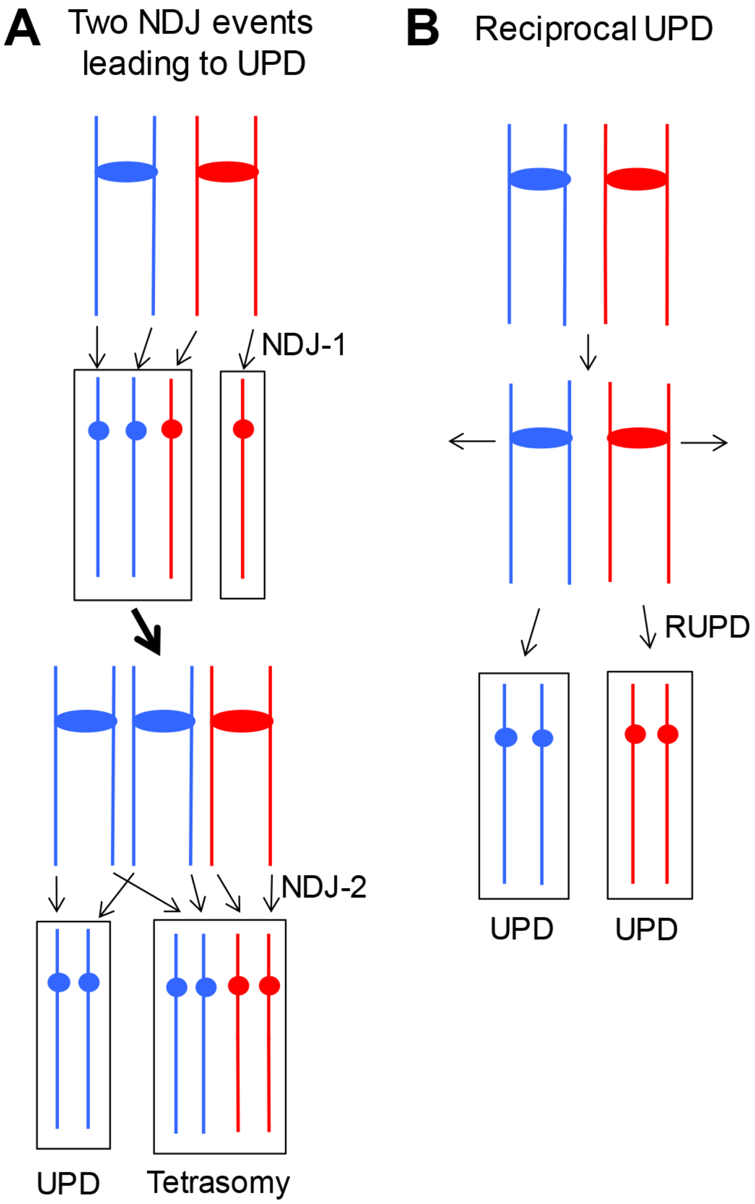

Supplementary Figure 7. Two pathways for generating UPD. (**A**) Two non-disjunction (NDJ) events in different cell cycles can result in UPD. NDJ-1 results in one trisomic strain and one monosomic strain. If a second NDJ event occurs in the trisomic strain, a UPD isolate can be generated. (**B**) Reciprocal UPD. By this mechanism, two NDJ events in the same cell cycle results in two daughter cells with reciprocal patterns of UPD.

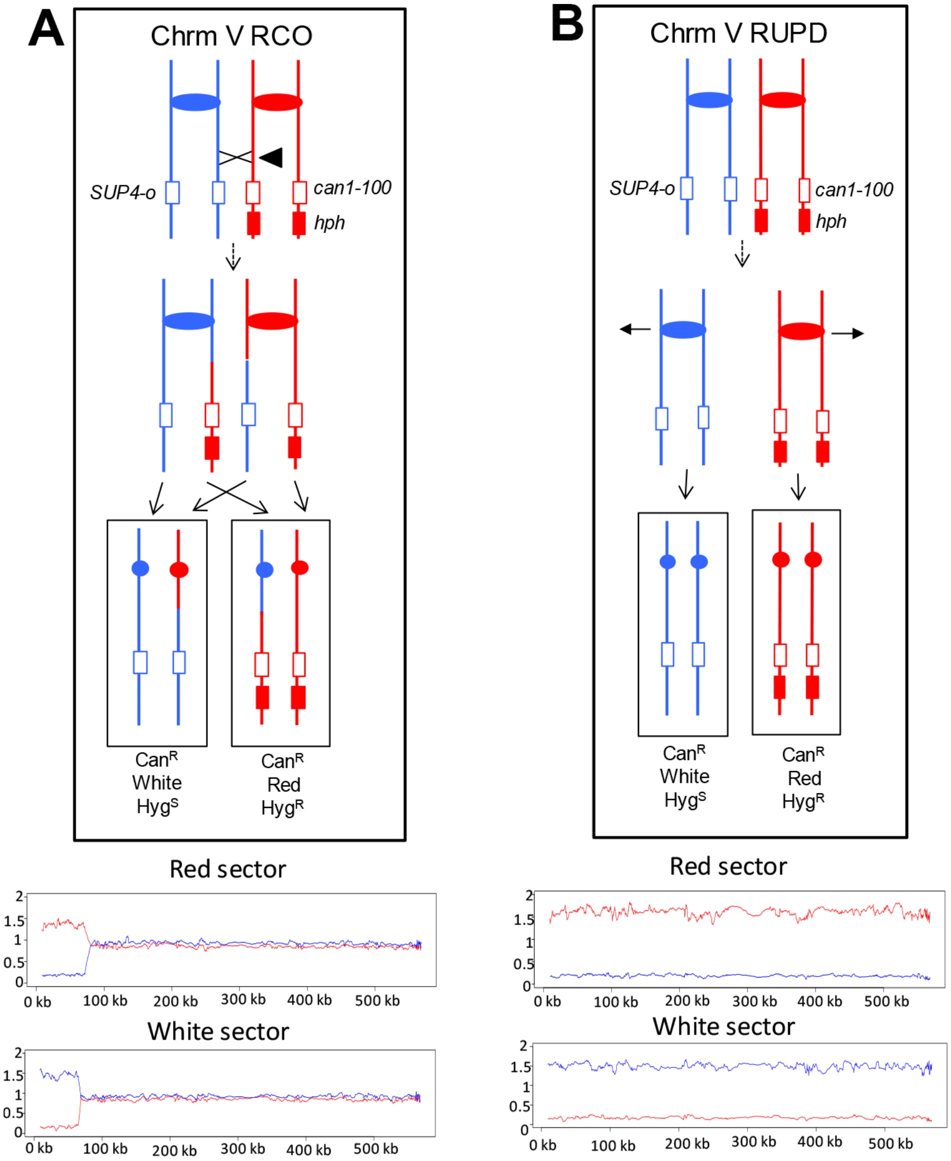

Supplementary Figure 8. Detection and analysis of reciprocal crossovers (RCO) and reciprocal UPD (RUPD). The chromosome V homolog derived from YJM789 (shown in blue) has a *SUP4-o* gene (encoding an ochre suppressor) replacing the *CAN1* gene. On the W303-1A-derived homolog, chromosome V has the ochre-suppressible *can1-100* allele with the *hph* gene (resulting in hygromycin resistance) located centromere-distal to *can1-100*. In addition, the strain is homozygous for the ochre-suppressible *ade2-1* allele; diploids with this gene and 0, 1, or 2 copies of *SUP4-o* form red, pink or white colonies, respectively. Diploids without a genetic alteration form pink, canavanine-sensitive, hygromycin-resistant colonies. (**A**) A reciprocal crossover between the *can1-100* marker and *CEN5* can result in a red/white Can^R sectored colony in which the white sector is sensitive to hygromycin. In the microarray pictures shown below the recombination event (FM1873-14 (E2) in Supplementary Table 2), hybridization values for chromosome V from the red and white sectors are shown on the top and bottom panels, respectively, at the bottom of the figure. (**B**) A reciprocal UPD event on chromosome V would be expected to produce a red/white Can^R sectored colony with the phenotypes identical to the RCO. The microarray patterns in each sector would be different. Microarrays derived from the red and white sectors of MD684.1.15 (E2) (Supplementary Table 2) are shown in the top and bottom panels, respectively, at the bottom of the figure.

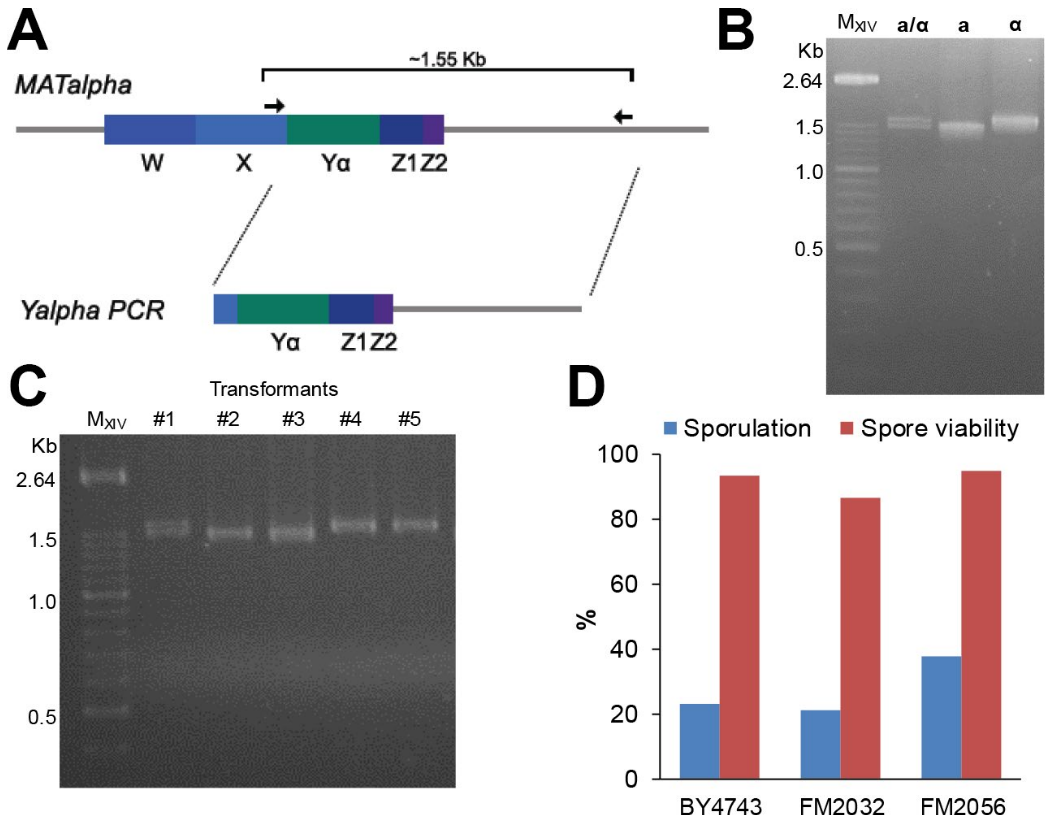

Supplementary Figure 9. One-step marker-free diploidization of *MAT*a *bar1Δ* strains. Proof of concept. (**A**) Schematic of the *MATα* locus and the PCR-based strategy to obtain the *MAT*a-to-*MATα* transformation product. The haploids *MAT*a and *MATα* differ in the Y sequence within the *MAT* locus (chromosome III), but shared flanking sequences. The Yα is ~100 bps longer than Ya (747 bps vs 642 bps). The forward primer X-reg (Supplementary Table 4) binds upstream of the Y region whereas the reverse primer MAT-R does so downstream, within the sequence adjacent to the MAT locus. Importantly, MAT-R does not bind downstream the silent *HMLα* and *HMR*a loci present in the same chromosome, which act during the mating type switching as template sequences for gene conversion at *MAT* [7]. (**B**) Gel separation of the three types of X-reg/MAT-R PCR products. The resulting PCR product is ~1.55 Kbps if the strain is a haploid *MATα*, whereas it is ~1.45 kb if the strain is a haploid *MAT*a strain. A diploid would yield a double band that can be resolved in a 2% agarose gel. In these examples, the haploids and the diploid are the widely used S288C reference strains BY4741, BY4742 and BY4743. (**C**) Testing transformants for the one-step marker-free diploidization of *MAT*a *bar1*Δ strains. Only *MAT*a strains respond to the α-factor pheromone with a transient G1 arrest. The arrest is relieved by the Bar1 protease, which degrades α-factor extracelullarly. Knock-out mutants for *BAR1* become highly hypersensitive to α-factor; hence, in *bar1*Δ strains, α-factor can be used for the selection against the haploid *MAT*a genotype. In this example, competent cells from the strain FM1932 (BY4741; *MAT*a Δ*bar1::URA3*) were transformed with the Yα PCR product and spread onto YPD plates supplemented with 5 μg α-factor (spread on the plate surface). Five colonies were picked, re-struck onto YPD α-factor plates, and their genomic DNA tested for the genomic content at the MAT locus by X-reg/MAT-R PCR. Note that clone #1 is a diploid and clones #4 and #5 have become haploid *MATα*. Clones #2 and #3 were still haploid *MAT*a. These strains probably come from spontaneous resistance to α-factor unrelated to genetic changes in the MAT locus (from our estimation: 0.2% over viable CFUs in a mock transformation experiment). (**D**) Sporulation efficiency and spore viability of the diploid obtained through the one-step marker-free method in comparison to diploids obtained through mating. BY4743 (*MAT*a/α *BAR1*/*BAR1*); FM2032 (control *MAT*a/α *bar1*Δ/*bar1*Δ obtained by mating the corresponding *bar1*Δ haploids); FM2056 (clone #1 from panel C).
