# Supplementary material for: Cytological and genetic consequences for the progeny of a mitotic catastrophe provoked by Topoisomerase II deficiency
Source: Aging (Albany NY). 2019 Dec 8;11(23):11686–721. doi: 10.18632/aging.102573 (PMC6932922; doi:10.18632/aging.102573)
Supplement: Supplementary Tables [file aging-11-102573-s002..pdf]

SUPPLEMENTARY TABLES

Supplementary Table 1. P-value of cross comparisons between the number of cell bodies generated after the 37 °C for 6 h → 25 ºC for 16 h regime.

Strain/condition	Microcolonies of ≥2 cell bodies at 37 ºC (N)	Microcolonies that rebudded after 25 ºC downshift (N)	P-value against *top2-5* (first row)
top2-5	160	26	-
top2-5 (Sorb)	89	21	0.1064
*top2-5 yca1*Δ	152	35	0.086
top2-5/top2-5	90	42	< 0.0001

Comparison between microcolonies of ≥2 cell bodies after the 37 °C incubation and re-budding (for at least one cell body) after the 25 °C downshift were performed in 2×2 contingency tables using a one-tailed Fisher's exact test.

Supplementary Table 2. Summary of chromosome V events in R HygR/W HygS sectored colonies of FM1873 and MD684 strains.

Strain name[1] and sector pattern[2]	Genomic alterations[3]
FM1873-1 (E2) R Hyg^R/P-W Hyg^S	UPD on V in "correct" direction in red sector; partial UPD in "incorrect" direction on V in white sector.
FM1873-2 (E2) R Hyg^R/W Hyg^S	UPD on V in "correct" direction in red sector; T-LOH on V in "correct" direction in white sector.
FM1873-4 (E2) R Hyg^R/W Hyg^S	RCO on V. Breakpoint at 134 kb in red sector and 144 kb in white sector.
FM1873-12 (E2) R Hyg^R/W Hyg^S	RUPD on V.
FM1873-14 (E2) R Hyg^R/W Hyg^S	RCO on V. Breakpoint at 67 kb in red sector and 76 kb in white sector.
FM1873-26 (E2) R Hyg^R/W Hyg^S	UPD in correct direction on V in red sector; T-LOH event in correct direction in white sector.
FM1873-32 (E2) R Hyg^R/P Hyg^S	RUPD on V
FM1873-35 (E2) R Hyg^R/P Hyg^S	RUPD on V
FM1873-37 (E2) R Hyg^R/P Hyg^S	RUPD on V
FM1873-41 (E2) R Hyg^R/P Hyg^S	RUPD on V
FM1873-45 (E2) R Hyg^R/P Hyg^S	UPD on V in the correct direction in the red sector (two copies of W303-1A-derived chromosome; white sector has one copy of YJM789-derived homolog and none of W303-1A-derived homolog
FM1873-49 (E2) R Hyg^R/P Hyg^S	No obvious changes on V in red sector; white sector has UPD on V in correct direction.
FM1873-50 (E2) R Hyg^R/P Hyg^S	RUPD on V
FM1873-54 (E2) R Hyg^R/P Hyg^S	RUPD on V
FM1873-70 (E2) R Hyg^R/P Hyg^S	RUPD on V
FM1873-85 (E2) R Hyg^R/P Hyg^S	RUPD on V
FM1873-101 (E2) R Hyg^R/P Hyg^S	RCO on V. Breakpoints at 95 kb and 86 kb in red sector. The red sector has event indicative of a G1-associated DSB, repaired in G2. Breakpoint at 93 kb in white sector.
FM1873-105 (E2) R Hyg^R/P Hyg^S	RUPD on V.
FM1873-106 (E2) R Hyg^R/P Hyg^S	RUPD on V.
FM1873-112 (E2) R Hyg^R/P Hyg^S	RUPD on V.
FM1873-1 (C2)	UPD on V in "correct" direction in red sector; partial UPD in "incorrect" direction on V in white sector.
FM1873-2 (C2)	UPD on V in "correct" direction in red sector; no clear event on V in white sector.
FM1873-3 (C2)	No detectable events on V.
FM1873-7 (C2)	No detectable events on V in red sector. In white sector, V has terminal LOH (about 110 kb).
FM1873-14 (C2)	RUPD on V.
FM1873-19 (C2)	UPD on V in red sector; no obvious change on V in white sector.

FM1873-20 (C2)	RUPD on V.
MD684.1.15 (E2)	RUPD (V)
MD684.1.17 (E2)	RUPD (V)
MD684.1.49 (E2)	RUPD (V)
MD684.1.61 (E2)	Red sector looked like haploid strain (all homologs derived from W303-1A with elevated signal, all derived from YJM789 with reduced signal. UPD on V in white sector
MD684.1.65 (E2)	Red sector looked like haploid strain (all homologs derived from W303-1A with elevated signal, all derived from YJM789 with reduced signal). In white sector: UPD on V
MD684.1.73 (E2)	Red sector looked like haploid strain (all homologs derived from W303-1A with elevated signal, all derived from YJM789 with reduced signal). In white sector: UPD on V
MD684.1.75 (E2)	Red sector looked like haploid strain (all homologs derived from W303-1A with elevated signal, all derived from YJM789 with reduced signal). In white sector, UPD on V.
MD684.1.83 (E2)	RUPD (V)
MD684.1.88 (E2)	Red sector looked like haploid strain (all homologs derived from W303-1A with elevated signal, all derived from YJM789 with reduced signal). In white sector, T-LOH on V (breakpoint at 56 kb).

[1] Parentheses after the strain name indicate whether the strain was experimental (E2, incubated for six hours at 37 ºC in liquid) or control (C2, not incubated at the restrictive temperature).
[2] Strains either treated at 37 ºC for six hours (E) or untreated (C) at the restrictive temperature were plated on solid medium containing canavanine. After colonies were formed, we purified cells derived from red and white sectors, and determined whether cells from these sectors were Hyg^R or Hyg^S (as indicated in the table). The *hph* marker was located distal to *can1-100* on the W303-1A-derived chromosome. Thus, a reciprocal crossover (RCO) or reciprocal UPD event would be expected to produce a hygromycin-resistant red sector, and a hygromycin-sensitive white sector. Code: T-LOH (terminal LOH event), I-LOH (interstitial LOH event), Tri (trisomy), UPD (uniparental disomy), RCO (reciprocal crossover), and RUPD (reciprocal uniparental disomy).
[3] The arrays for sectored colonies were done with arrays that had dense SNPs for chromosomes I, III, V and VIII, but few SNPs on other chromosomes. Thus, we tabulate only events involving chromosome V.

Supplementary Table 3. Strains used in this work.

Strain name	Relevant genotype[a]	Origin
CH326	(S288C) *MAT*a *ura3-52 his4-539am lys2-801am SUC2+ top2-5*	D. Botstein[b]
CH335	(S288C) *MAT*a *ura3-52 his4-539am lys2-801am SUC2+ TOP2*	D. Botstein[b]
FM1386	CH326; *H2A2(YBL003c):GFP:BleMX*; Δ*bar1::URA3*	F. Machin[c]
FM1419[e]	CH335; *H2A2(YBL003c):GFP:BleMX*; Δ*bar1::URA3*	F. Machin[c]
FM1856	FM1386; Δ*yca1::kanMX4*	This work
FM1871[e]	FM1419; Δ*yca1::kanMX4*	This work
FM1730[f]	*MAT*a/α *top2-5/top2-5* homozygous diploid (from FM1386)	This work
FM1732[e,f]	*MAT*a/α *TOP2/TOP2* homozygous diploid (from FM1419)	This work
PSL2	(W303a) *MAT*a *ade2-1 can1-100 his3-11,15 ura3-1 trp1-1 V9229::HYG V261553::LEU2 RAD5*	T. Petes[d]
PSL5	(YJM789) *MAT*α *ade2-1 ura3 can1*Δ*::SUP4-o gal2 ho::hisG*	T. Petes[d]
FM1830[g]	PSL2; *top2-5:9myc:natMX* (1-2 x cXIV)	This work
FM1832	PSL5; *top2-5:9myc:natMX*	This work
FM1873[g]	(FM1830 x FM1832) *MAT*a/α *top2-5/top2-5* hybrid diploid (3-4 x cXIV, cXIIr t-LOH)	This work
FM2010	(PSL2 x PSL5) *MAT*a/α *TOP2/TOP2* hybrid diploid	This work
MD681	PSL2 *top2-5:9myc:natMX* (FM1830 backcrossed with W303 to have 1 x cXIV)	This work
MD684	(MD681 x FM1832) *MAT*a/α *top2-5/top2-5* hybrid diploid (3-4 x cXIV)	This work
BY4743	*MAT*a/α *his3*Δ*1/his3*Δ*1 leu2*Δ*0/leu2*Δ*0 met15*Δ*0/MET15 LYS2/lys2*Δ*0 ura3*Δ*0/ura3*Δ*0*	Euroscarf collection
FM1932	(BY4741) *MAT*a *his3*Δ*1 leu2*Δ*0 met15*Δ*0 ura3*Δ*0*; Δ*bar1::URA3*	This work
FM1982	(BY4742) *MAT*a *his3*Δ*1 leu2*Δ*0 lys2*Δ*0 ura3*Δ*0*; Δ*bar1::URA3*	This work
FM2032	(FM1932 x 1982) *MAT*a/α *bar1*Δ*/bar1*Δ	This work
FM2056[f]	(clone #1 in Supplementary Figure9C) *MAT*a/α *bar1*Δ*/bar1*Δ (from FM1932)	This work

[a] Semicolons separate independent transformation events during strain construction. Intermediate strains are omitted.
[b] Described in [8]
[c] Described in [5]
[d] Described in [3]
[e] These strains were used as *TOP2* controls during clonogenic assays (n=3 independent experiments). In all cases, 100% viability was maintained after 0, 3, 6, 9, 12, 24 and 48 h incubations at 37 ºC.
[f] These homozygous diploids were made through the one-step marker-free transformation-based protocol described in Supplementary Figure 9.
[g] These strains were shown by SNP and copy number arrays to carry the genome alteration shown between brackets. For instance, the hybrid heterozygous *top2-5/top2-5* diploid FM1873 carried two genome rearrangements when compared to its isogenic *TOP2/TOP2* counterpart: 3-4 copies of cXIV and a t-LOH at cXII right arm.

Supplementary Table 4. Primers used in this study.

Primer name	Purpose	Sequence (5′ to 3′)
Yca1-F (-359)	To amplify Δ*yca1::kanMX* from gDNA	CAATGCATTGGATCTTATTGGC
Yca1-R (+1709)	To amplify Δ*yca1::kanMX* from gDNA	GTCGAAACAAGAAGAGCAAAC
Bar1-F (-196)	To amplify Δ*bar1::URA3* from gDNA	GCCAGCTATTCTGAAACACACCAC
Bar1-R (+2316)	To amplify Δ*bar1::URA3* from gDNA	AACAGTCTTAGGGAAGTAACGAG
Top2-S3	To tag *TOP2* at 3' with *9xmyc:natMX*	GGAAAACCAAGGATCAGATGTTTCGTTCAATGAAGAGGATCGTACGCTGCAGGTCGAC
Top2-S2	To tag *TOP2* at 3' with *9xmyc:natMX*	TATAAAAAGAATGGCGCTTTCTCGGATAAATATTATTCAATCGATGAATTCGAGCTCG
Top2-F (-175)	To amplify top2-5*:9xmyc:natMX* from gDNA	AAGACGCGCCAGTAGGACGC
Top2-R (+4511)	To amplify top2-5*:9xmyc:natMX* from gDNA	CGCACGATGTTTTTCGCCCAGG
Xreg-F	To amplify Yα region in the MAT locus (Yα transformation product)	TTGTTGGCCCTAGATAAGAA
MAT-R (+2894)	To amplify the MAT locus (Yα transformation product)	CAAGGGAGAGAAGACTTGTG

Supplementary Table 5. Landscape of possible outcomes during microcolony experiments.

After 37 ºC (Top2 inactivation)	After 25 ºC reincubation (Top2 re-activation)
0 (lysis)	---
1 (did not bud)	0 (lysis)
	1 (remained unbudded)
	2 (able to bud once Top2 is back)
	3, 4, 5, etc. (short-term budding capability)
	>20-50 (will raise a viable population)
2 (did bud once without Top2)	0 (double lysis)
	1 (one body lysed; the other did not divide again)
	2 (no more budding even after Top2 reactivation)
	3 (one body able to bud once Top2 is back)
	4, 5, etc. (both bodies able to bud*)
	>20-50 (at least one body/cell is viable)
3 (did bud twice without Top2)	0, 1, 2 (no more budding and some bodies lysed)
	3 (no more budding even after Top2 reactivation)
	4 (1 of 3 bodies rebudded once)
	5 (2 of 3 bodies rebudded once*)
	6, etc. (3 of 3 bodies rebudded once *)
	>20-50 (at least one body/cell is viable)
4 (mother and daughter rebudded again*)	0, 1, 2, 3 (no more budding and some bodies lysed)
	4 (no more budding even after Top2 reactivation)
	5, 6, 7, 8, 9 (1-4 of 4 bodies rebudded once*)
	>20-50 (at least one body/cell is viable)
Etc.	Etc.

* Other interpretations on the origin of these microcolonies are possible.
